# Transdermal Transfersome Nanogels Control Hypertrophic Scar Formation via Synergy of Macrophage Phenotype‐Switching and Anti‐Fibrosis Effect

**DOI:** 10.1002/advs.202305468

**Published:** 2023-12-08

**Authors:** Yunsheng Chen, Kun Chen, Shan Zhong, Jiaqiang Wang, Zhixi Yu, Xiyang Sun, Yue Wang, Yan Liu, Zheng Zhang

**Affiliations:** ^1^ Department of Burn Shanghai Burn Institute Ruijin Hospital Shanghai Jiao Tong University School of Medicine 197 Ruijin 2nd Road Shanghai 200025 China; ^2^ Department of Burn and Plastic Surgery Beijing Children's Hospital Capital Medical University National Center for Children's Health Beijing 100045 China; ^3^ Shunyi Maternal and Children's Hospital of Beijing Children's Hospital Beijing 101300 China; ^4^ Department of Plastic and Reconstructive Surgery Shanghai Ninth People's Hospital School of Medicine Shanghai Jiao Tong University 639 Zhizaoju Rd Shanghai 200011 China; ^5^ Hongqiao International Institute of Medicine Tongren Hospital School of Medicine Shanghai Jiao Tong University 1111 XianXia Road Shanghai 200336 China; ^6^ Department of Ear Reconstruction Plastic Surgery Hospital Chinese Academy of Medical Sciences and Peking Union Medical College 33 Badachu Road Beijing 100144 China

**Keywords:** anti‐fibrosis effect, hypertrophic scar formation, macrophage phenotype‐switching, transdermal delivery, transfersome nanogels

## Abstract

Hypertrophic scar (HS), which results from prolonged inflammation and excessive fibrosis in re‐epithelialized wounds, is one of the most common clinical challenges. Consequently, sophisticated transdermal transfersome nanogels (TA/Fu‐TS) are prepared to control HS formation by synergistically inhibiting inflammation and suppressing fibrosis. TA/Fu‐TSs have unique structures comprising hydrophobic triamcinolone acetonide (TA) in lipid multilayers and hydrophilic 5‐fluorouracil in aqueous cores, and perform satisfactorily with regard to transdermal co‐delivery to macrophages and HS fibroblasts in emerging HS tissues. According to the in vitro*/*vivo results, TA/Fu‐TSs not only promote macrophage phenotype‐switching to inhibit inflammation by interleukin‐related pathways, but also suppress fibrosis to remodel extracellular matrix by collagen‐related pathways. Therefore, TA/Fu‐TSs overcome prolonged inflammation and excessive fibrosis in emerging HS tissues, and provide an effective therapeutic strategy for controlling HS formation via their synergy of macrophage phenotype‐switching and anti‐fibrosis effect.

## Graphical abstract

Transfersome nanogels (TA/Fu‐TS) realize the transdermal co‐delivery of hydrophobic triamcinolone acetonide and hydrophilic 5‐fluorouracil to macrophages and fibroblasts in emerging hypertrophic scars (HS). Then, TA/Fu‐TSs promote macrophage phenotype‐switching to inhibit inflammation and suppress fibrosis to remodel extracellular matrix. Therefore, TA/Fu‐TSs overcome prolonged inflammation and excessive fibrosis in emerging HS tissues, and provide an effective strategy for controlling HS formation.

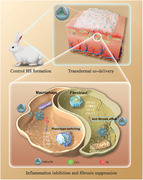

## 1 Introduction

Hypertrophic scar (HS) is a pathological symptom of excessive fibrosis, and it often results in various physical and psychological problems for patients.^[^ ^1^ ^]^ However, conventional therapeutic approaches, such as surgical resection and steroid injections, have some disadvantages including high recurrence and painful procedures.^[^ ^2^ ^]^ Once HS formation finished, it presents a significant clinical challenge. Recently, attention has been focused on early intervention in re‐epithelialized wounds to control HS formation, and it has proved to be a feasible approach for HS treatment.^[^ ^3^ ^]^ Thus, it is essential to understand the fundamental mechanism by which HS occurs, and to establish an effective strategy for controlling HS formation. According to various hypotheses on pathogenesis, excessive fibrosis is responsible for HS, and inflammation is considered a trigger.^[^ ^4^ ^]^ Although inflammation is essential for promoting wound healing, prolonged inflammation results in persistent excessive fibrosis, which produces excessive extracellular matrix (ECM) in emerging HS tissues.^[^ ^5^ ^]^ Therefore, an ideal strategy for controlling HS formation is to synergistically inhibit inflammation and suppress fibrosis.

Macrophage phenotypic switching has attracted attention as a potential approach for inhibiting inflammation.^[^ ^6^ ^]^ Macrophages, which are inflammatory cells, are essential for wound healing and divided into a pro‐inflammatory phenotype (M1, which causes inflammation through the secretion of pro‐inflammatory cytokines) and an anti‐inflammatory phenotype (M2, which suppresses inflammation by secreting anti‐inflammatory cytokines).^[^ ^7^ ^]^ Promoting macrophage phenotype‐switching from M1 to M2 is regarded as a valuable strategy for inhibiting inflammation in various diseases. In particular, triamcinolone acetonide (TA), a synthetic glucocorticoid, is recommended in clinical practice for anti‐inflammation by inducing differentiation toward M2 phenotype.^[^ ^8^ ^]^ In the aspect of fibrosis suppression, 5‐fluorouracil (5‐Fu) has been proven to possess anti‐fibrosis effect to blockade collagen synthesis by inhibiting TGF‐β signaling pathway.^[^ ^9^ ^]^ Therefore, a combination of TA and 5‐Fu seems an effective strategy to control HS formation by integrating inflammation inhibition and fibrosis suppression.

Although subcutaneous injection of TA and 5‐Fu is the first‐line modality in HS treatment, it is limited in emerging HS tissues owing to the difficulty of application and poor cellular uptake.^[^ ^10^ ^]^ Recently, the emergence of nanotechnology has opened up opportunities in medicine.^[^ ^11^ ^]^ Nanotechnology‐based transdermal delivery is a facile approach to ensuring effective cellular uptake.^[^ ^12^ ^]^ Therefore, suitable nanocarriers for the transdermal co‐delivery of hydrophobic TA and hydrophilic 5‐Fu are necessary for controlling HS formation. In our prior works, nanoethosomes have investigated as transdermal nanocarriers because they have flexible phospholipid bilayers.^[^ ^13^ ^]^ However, they are unsuitable for hydrophobic TA owing to their monolayer structures. In this regard, transfersomes (TS), the nanovesicles composed of phospholipid multilayers and aqueous cores, are designed for the co‐delivery of TA and 5‐Fu.^[^ ^14^ ^]^ Furthermore, TS have malleable structure to improve their penetration ability because of the action of surfactants. Hence, with the unique structure of TA in lipid multilayers and 5‐Fu in aqueous cores, the prepared TS own the function of transdermal co‐delivery to control HS formation.

**Scheme 1:**
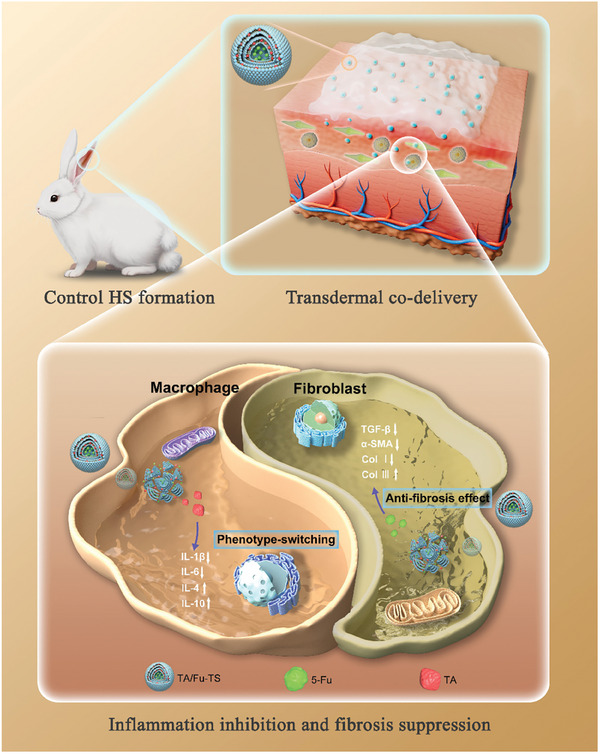
Schematic illustration of TA/Fu‐TS in transdermal co‐delivery and controlling HS formation via synergy of macrophage phenotype‐switching and anti‐fibrosis effect.

Herein, sophisticated TA and 5‐Fu co‐loaded TS nanogels (TA/Fu‐TS) are prepared to control HS formation via synergy of macrophage phenotype‐switching and anti‐fibrosis effect. Benefiting from their unique structure of TA in lipid multilayers and 5‐Fu in aqueous cores, TA/Fu‐TS enable the co‐delivery of TA and 5‐Fu to macrophages and HS fibroblasts (HSF) in emerging HS tissues. According to the in vitro*/*vivo results, TA/Fu‐TS synergistically inhibit inflammation and suppress fibrosis to control HS formation (**Scheme** **1**). Therefore, TA/Fu‐TS overcome prolonged inflammation and excessive fibrosis, and provide an effective therapeutic strategy for controlling HS formation via the synergy of macrophage phenotype‐switching and anti‐fibrosis effect.

## 2 Result and Discussions

### 2.1 Characterization Studies

**Figure 1:**
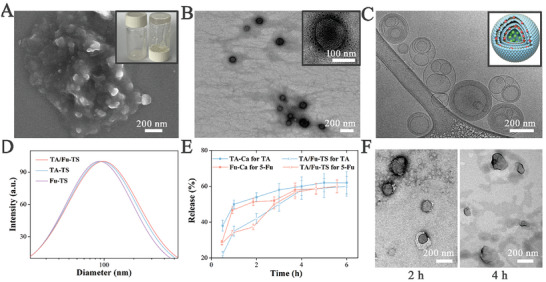
A) SEM image of TA/Fu‐TS (Insert: the photo of nanogels); B) TEM image of TA/Fu‐TS (Insert: the detail of TA/Fu‐TS); C) Cryo‐TEM image of TA/Fu‐TS (Insert: the schematic of TA/Fu‐TS); D,E) Size distributions and release profiles; F) TEM images of degraded TA/Fu‐TS.

The morphological features of TA/Fu‐TS were studied by electron microscopy. According to a topography effect in scanning electron microscopy (SEM) image, spherical vesicles were clearly visible and densely distributed in gel matrix, indicating TA/Fu‐TS nanogels exhibited sufficient adhesion to the skin and achieved transdermal delivery (**Figure** **1**A). Transmission electron microscopy (TEM) images also confirmed that TA/Fu‐TS nanogels were composed of intact spherical lamellar vesicles. More specifically, the lamellae extended to the core of the vesicles, which matched with the characteristics of the TS (Figure 1B). In cryogenic TEM (Cryo‐TEM) image, homogeneous TA/Fu‐TS exhibited the multilayered structures surrounding the aqueous core, which was favored for the loading of hydrophobic TA in lipid multilayered and hydrophilic 5‐Fu in aqueous core (Figure 1C).

The physic‐chemical features, including size distribution, release profile, and entrapment efficiency, were further studied. Compared with Fu‐TS (115 ± 43 nm), TA/Fu‐TS and TA‐TS exhibited the larger diameters (127 ± 57 and 124 ± 49 nm), due to the immobilization of TA in lipid multilayers (Figure 1D). In addition, the release profile and entrapment efficiency were studied using dialysis method. Considering the drugs unloaded by TS, the mixtures of drug and Carbopol (named TA‐Ca and Fu‐Ca) were introduced as control. All groups exhibited the fast release in 1 h, indicating they had released the most of drugs unloaded in TS. In contrast, TA/Fu‐TS had slow releases between 1 and 2 h, and accelerated releases between 2 and 5 h (Figure 1E). Meanwhile, the release was monitored by TEM (Figure 1F). After 2 h, TEM revealed that the drugs had barely leaked from the intact TA/Fu‐TS. After 4 h, the multilayered structure ruptured, and TA was released owing to the degradation of the lipid multilayers. Therefore, the entrapment efficiency was calculated as the release amount in 2–5 h, which was 17% ± 3.4% for TA and 24% ± 3.5% for 5‐Fu.

In summary, TA/Fu‐TS presented a unique structure of TA in lipid multilayers and 5‐Fu in aqueous cores, and has potential as a transdermal co‐delivery system for the control of HS.

### 2.2 Cell Studies

#### 2.2.1 Cytotoxicity Evaluation

**Figure 2:**
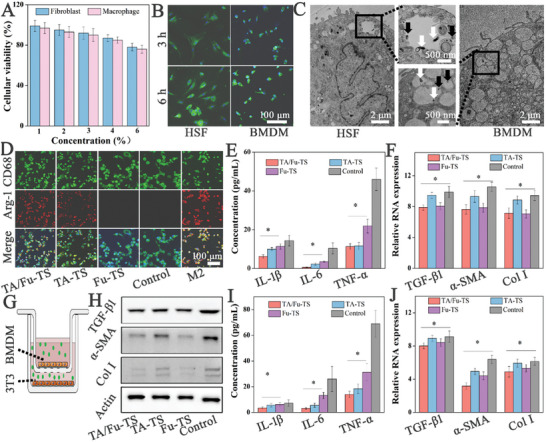
A) The cellular viability of HSF and BMDM treated TA/Fu‐TS; B) CLSM images of HSF and BMDM treated TA/Fu‐TS; C) Ultrastructural observation of HSF and BMDM treated TA/Fu‐TS (white and black arrows: intact and cracked TA/Fu‐TS); D) CLSM images of macrophage phenotype‐switching; E,F) The biomarkers of inflammation inhibition and fibrosis suppression in mono‐culture models; G) Schematic of co‐culture models; H) Fibrosis‐related protein expressions in co‐culture models; and J,I) The biomarker detection of inflammation inhibition and fibrosis suppression in co‐culture models.

The cytotoxicity of TA/Fu‐TS was crucial for their potential applications. Cell counting kit‐8 (CCK‐8) assay revealed that TA/Fu‐TS exhibited a concentration‐dependent cytotoxicity in HSF and bone marrow‐derived macrophages (BMDM) (**Figure** **2**A). No significant cytotoxicity was observed at TA/Fu‐TS concentrations of 1% or 2% (the cellular viability >90%). At a concentration of 3%, TA/Fu‐TS reduced the viability of the BMDMs (the cellular viability <90%). However, there was a slight decrease in the viabilities of both HSF and BMDMs (<85%) at TA/Fu‐TS concentration of 4% and 5%. Therefore, TA/Fu‐TS exhibited excellent biocompatibility, and a concentration of 2% was used in subsequent studies.

#### 2.2.2 Cellular Uptake Evaluation

Cellular uptake was evaluated using fluorescence‐labeled TA/Fu‐TS. In confocal laser scanning microscopy (CLSM) images, the fluorescence indicated the profiles of both HSF and BMDMs, confirming that the cellular uptake of TA/Fu‐TS was not affected by their multilayered structure (Figure 2B). Flow cytometry also revealed the satisfactory cellular uptake of TA/Fu‐TS by both BMDM and HSF (Figure S1, Supporting Information). More details of cellular uptake were further investigated by TEM (Figure 2C). The intact TA/Fu‐TS in cytoplasm (indicated by white arrows) revealed efficient transmembrane delivery, and the fraction of TA/Fu‐TS in the cytoplasm (indicated by black arrows) also indicated that TA/Fu‐TS realized the release of TA and 5‐Fu. Therefore, TA/Fu‐TS could facilitate the co‐delivery of TA and 5‐Fu to both BMDM and HSF, which was favor to inhibit inflammation and suppress fibrosis.

#### 2.2.3 Induction of Macrophage Phenotype‐Switching

As the increase of M2 phenotype could moderate inflammation to control HS formation, the efficiency of TA/Fu‐TS inducing macrophage phenotype‐switching was investigated with CD68 and Arg‐1 (Figure 2D).^[^ ^15^, ^16^ ^]^ TA/Fu‐TS and TA‐TS increased Arg‐1 fluorescence compared to that in the Fu‐TS and M2 phenotype groups, indicating TA played the key role in macrophage phenotype‐switching as expected. Thus, TA/Fu‐TS effectively induced macrophage phenotype‐switching from M1 to M2 phenotype, and the increase of M2 phenotype was favored to inhibit inflammation.

#### 2.2.4 Inflammation Inhibition and Fibrosis Suppression

Following macrophage phenotype‐switching, the inflammation inhibition and fibrosis suppression were studied by detecting related biomarkers in both mono‐ and co‐culture models. The enzyme‐linked immunosorbent assay (ELISA) investigations revealed a significant reduction in pro‐inflammatory cytokines in the mono‐cultures of BMDMs with TA/Fu‐TS and TA‐TS, which matched with the increase of M2 phenotype (Figure 2E). Similar, fibrosis was suppressed in the monocultures of HSF with TA/Fu‐TS and Fu‐TS, due to the anti‐fibrosis effect of 5‐Fu as expected (Figure 2F). Subsequently, the co‐culture of BMDM and 3T3 (established fibroblasts lines of mouse origin) was further carried out to assess the synergy between macrophage phenotype‐switching and anti‐fibrosis effect as schematic presentations (Figure 2G). TA/Fu‐TS performed best with regard to inhibiting inflammation and suppressing fibrosis (Figure 2H,J,I). Interestingly, the western blotting and quantitative polymerase chain reaction (qPCR) results showed that TA‐TS was effective in suppressing fibrosis, suggesting the synergy of macrophage phenotype‐switching and anti‐fibrosis effect that suppressed fibrosis more effectively.

In summary, TA/Fu‐TS could co‐deliver drugs to macrophages and HSF, and provide the best performance in fibrosis suppression with the synergy of macrophage phenotype‐switching activity of TA and anti‐fibrosis activity of 5‐Fu.

### 2.3 Transdermal Co‐Delivery Study

**Figure 3:**
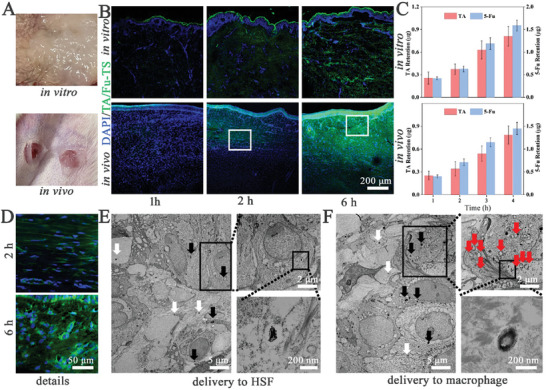
A) Schematic of in vitro*/*vivo transdermal delivery; B) CLSM images of HS in vitro*/*vivo administered with TA/Fu‐TS; C) Retention amounts of TA and 5‐Fu in HS tissues after in vitro*/*vivo admonition; D) Penetration details of TA/Fu‐TS; E,F) TEM image of TA/Fu‐TS transdermal delivering to HSF and macrophage (white and black arrows: TA/Fu‐TS in ECM and cytoplasm, red arrows: phagosome).

The transdermal co‐delivery of TA/Fu‐TS was the primary concern in controlling HS formation, and was evaluated using NBD‐labeled formulation nanogels (green fluorescence) applied to human HS tissues (in vitro) and rabbit HS models (in vivo) as schematic presentations (**Figure** **3**A). The time‐dependent increase in fluorescence distribution revealed by the in vitro*/*vivo studies suggested that TA/Fu‐TS could achieve the transdermal permeation into human and rabbit HS tissues (Figure 3B). Compared to traditional liposomes, the TA/Fu‐TS exhibited the better penetration ability, because ethanol and sodium deoxycholate endowed them with malleable structures.^[^ ^17^ ^]^ Furthermore, the time‐dependent increase in the retention of TA and 5‐Fu provided more evidence that the TA/Fu‐TS were capable of transdermal co‐delivery into HS tissues (Figure 3C). After amplifying of the zone of the white frame, the co‐localization of green and blue fluorescence indicated that TA/Fu‐TS had effectively entered and accumulated in the cells of the HS tissue (Figure 3D). The transdermal co‐delivery to HSF and macrophage in vivo was further verified by TEM. Intact TA/Fu‐TS (indicated by white arrows) were found in the dermis, and their multilayer and large core facilitated penetration into dermis via their deformable membrane through extracellular penetration pathway. Furthermore, TA/Fu‐TS (indicated by black arrows) were also found in cytoplasm, indicating that they had effectively entered the cells in dermis, principally HSF (Figure 3E). Interestingly, the numerous phagosome (indicated by red arrows) also suggested that TA/Fu‐TS entered macrophages in vivo (Figure 3F).^[^ ^18^ ^]^ Therefore, based on these results, TA/Fu‐TS could achieve the transdermal co‐delivery of TA and 5‐Fu to macrophages and HSF in HS tissues, which facilitated macrophage phenotype‐switching and inhibited fibrosis.

### 2.4 In Vivo Efficacy of Controlling HS Formation

**Figure 4:**
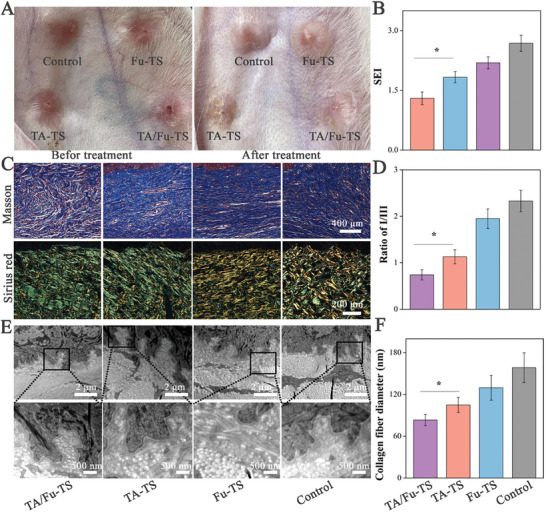
A) The appearance changes of HS; B) Statistics of SEI after treatment (*: *p*<0.05); C) Masson and Sirius red staining; D) Statistical analysis of the ratio of Col I to Col III (*: *p*<0.05); D,E) Ultra‐features of collagen in treated HS tissues; F) Statistical analysis of the collagen diameter in treated HS tissues.

The ability of TA/Fu‐TS to control HS formation was investigated by applying in rabbit HS models. Before treatment (following wound re‐epithelialization), emerging HS tissues appeared dark‐red. After treatment for 1 month, HS tissues in TA/Fu‐TS group exhibited the minimal redness and softness (**Figure** **4**A). Scar elevation index (SEI) also indicated that TA/Fu‐TS significantly decreased the scar thickness compared to that in other groups, further suggesting that the synergy of macrophage phenotype‐switching and anti‐fibrosis effect played key role in controlling HS formation (Figure 4B). Collagen deposition, the indicator of ECM remodeling, was evaluated histologically using Masson and Sirius red staining (Figure 4C). Masson's trichrome staining revealed numerous bulky and disorganized collagen formations in Control group. In contrast, TA/Fu‐TS treatment achieved the greatest improvement in slender collagen formation, with regular and parallel arrangements, compared to treatment with the other formulations. Sirius red staining was used to evaluate the collagen composition of HS tissues (yellowish red for Col I, green for Col III). The I/III ratio strongly indicated that TA/Fu‐TS was most effective at increasing the Col III content, as expected (Figure 4D). Moreover, the details of collagen deposition were studied using TEM (Figure 4E). Similar as histological analysis, collagen was disorganized in Control group, whereas formulations promoted collagen remodeling into interwoven collagen fibrils. TA/Fu‐TS ameliorated HS with thinner collagen and more regular arrangement, which was in accordance with the softened HS tissue (Figure 4F).^[^ ^19^ ^]^ Therefore, these results demonstrated the efficacy of TA/Fu‐TS with regard to control HS formation by improving ECM remodeling.

### 2.5 Macrophage Phenotype‐Switching and Inflammation Inhibition In Vivo

**Figure 5:**
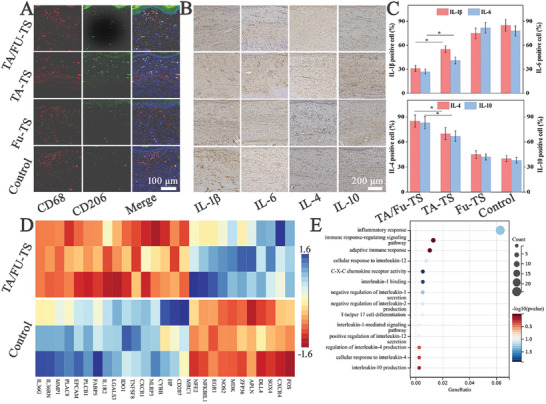
A) CLSM images of macrophage phenotype‐switching in vivo; B,C) Immunohistochemical and statistical analysis of inflammation‐related biomarkers (*: *p*<0.05); D) Heatmap of the top inflammation‐related DEGs after being further screened (HS tissues as RNA‐seq samples, *n* = 3); E) GO of the DEGs showing the enriched mainly altered molecular events.

To investigate the mechanisms underlying TA/Fu‐TS controlling HS formation, macrophage phenotype‐switching and inflammation inhibition were analyzed using immunofluorescence and immunohistochemical analysis, respectively. TA/Fu‐TS presented the most significant effect on enhancing M2 phenotype in HS tissues, suggesting that they could ameliorate inflammation (**Figure** **5**A). Subsequently, the immunohistochemical and statistical analyses indicated that TA/Fu‐TS was best at decreasing pro‐inflammatory cytokines (IL‐1β and IL‐6) and increasing anti‐inflammatory cytokines (IL‐4 and IL‐10) (Figure 5B,C). To further understand the molecular mechanism by which TA/Fu‐TS inhibited inflammation, total RNA was extracted from HS tissues and sequenced for comparison to differential expression genes (DEG). The heatmap of inflammation‐related DEG revealed that TA/Fu‐TS group had 17 upregulated and 11 downregulated DEGs compared to Control group (Figure 5D, criteria: *P* < 0.05, fold change >1.5). GO analysis demonstrated that the interleukin cytokines were the major molecular event altered in TA/Fu‐TS inhibiting inflammation (Figure 5E).^[^ ^20^ ^]^ Therefore, TA/Fu‐TS could promote the macrophage phenotype‐switching, and inhibit inflammation by interleukin‐related pathways.

### 2.6 Fibrosis Suppression In Vivo

**Figure 6:**
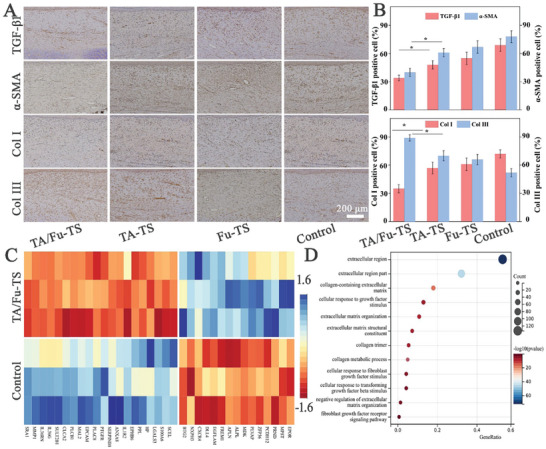
A,B) Immunohistochemical and statistical analysis of fibrotic biomarkers; (*: *p*<0.05); C) Heatmap of the top fibrosis‐related DEGs after being further screened (HS tissues as RNA‐seq samples, *n* = 3); D) GO of the DEGs showing the enriched mainly altered molecular events.

Fibrosis played the key role in controlling HS formation, and it was assessed by immunohistochemical analysis of fibrosis related protein expression (**Figure** **6**A,B). The results indicated that TA/Fu‐TS were best at suppressing the expression of TGF‐β1 and α‐SMA, indicating they could reduce the deposition of ECM. TA/Fu‐TS demonstrated the improved efficacy in reducing Col I and augmenting Col III, suggesting they could improve the quality of ECM. To elucidate the transcriptomic changes associated with fibrosis suppression by TA/Fu‐TS, the DEG was further subjected to RNA sequencing. The heatmap of fibrosis‐related DEG revealed that TA/Fu‐TS group had 20 upregulated and 15 downregulated DEGs compared to Control group (Figure 6C, criteria: *p* < 0.05, fold change >2). GO analysis demonstrated that TA/Fu‐TS could suppress fibrosis and improve ECM remodeling through pathways related to collagen synthesis and degradation (Figure 6D).^[^ ^21^ ^]^ Interestingly, in the result of DEG and GO analysis between TA‐TS and Control, TA could cause fibrosis suppression via macrophage phenotype‐switching. The similar result also revealed that Fu‐TS could inhibit inflammation via an‐fibrosis effect (Figure S2, Supporting Information). Therefore, TA/Fu‐TS exhibited great potential to suppress fibrosis to improve ECM remodeling via their synergy of macrophage phenotype‐switching and anti‐fibrosis effect. They also exhibited the satisfactory performance in controlling scar formation by overcoming prolonged inflammation and excessive fibrosis.

## 3 Conclusion

The sophisticated TA/Fu‐TS achieved the transdermal co‐delivery of TA and 5‐Fu to macrophage and HSF in emerging HS tissue. TA/Fu‐TS not only promoted the macrophage phenotype‐switching to inhibit inflammation via interleukin‐related pathways, but also suppressed fibrosis through the pathways related to collagen synthesis and degradation. Therefore, TA/Fu‐TS were proven to be control HS formation by overcoming prolonged inflammation and excessive fibrosis in cell and animal models. Thus, the present study provided an effective therapeutic strategy for controlling HS formation via the synergy of macrophage phenotype‐switching and anti‐fibrosis effect. Future studies are required to provide additional experimental evidence to support the clinical application of TA/Fu‐TS.

## 4 Experimental Section

### Preparation of TA/Fu‐TS

TA/Fu‐TS were prepared by film hydration technique followed by sonication. The organic phase was prepared by dissolving TA (1.2 mg), phosphatidylcholine (200 mg, soybean lecithin with 95.8%), and sodium deoxycholate (40 mg) in the mixture of chloroform and methanol (2:1, v/v, 5 mL). The aqueous phase was obtained by dissolving 5‐Fu (5 mg) in phosphate buffer (pH 7.4, 10 mL, containing 10% ethanol). A thin film was formed after removal of solvent under vacuum using a rotary evaporator at 40 °C. Then, TA/Fu‐TS dispersion was obtained by hydration of the thin film in aqueous phase, ultrasonic dispersion, and extrusion through the membrane (pore size of 100 nm). Finally, TA/Fu‐TS dispersion was mixed with Carbopol to prepare TA/Fu‐TS nanogels using a previously described protocol (S1, Supporting Information). NBD‐phosphatidylcholine was used to fluorescence labeling TS. Meanwhile, TA loaded TS (TA‐TS) and 5‐Fu loaded TS (Fu‐TS) were prepared by a method similar to that described above.

### Characterization of TA/Fu‐TS

TA/Fu‐TS was examined by TEM (JEM‐2010 JEOL, Japan, accelerating voltage of 120 KV), SEM (JSM‐6360LA, JEOL, Japan, accelerating voltage of 5 KV), and Cryo‐TEM (Talos F200C G2, Thermo fisher, MA, accelerating voltage of 200 KV). Size distribution was determined by dynamic light scattering (NiComp 380ZLS) at 25 °C. The in vitro release studies were evaluated according to the dialysis method and high performance liquid chromatography (HPLC) according to previous studies (the details in S2, Supporting Information).^[^ ^22^ ^]^

### Cell Culture

A common method was used in the isolation and culture of HSF from the fresh HS tissues (the ethical guidelines of the 1975 Declaration of Helsinki, the approval of Shanghai Ninth People's Hospital, SH9H‐2022‐TK332‐1). The HSF were collected and grown in DMEM containing fetal bovine serum (FBS, 10%) at 37 °C with 5% CO_2_. BMDM was isolated from femurs and tibias of C57/BL6 mice (4–6 weeks old, approval from the Animal Experimentation Ethics Committee of School of Medicine, Shanghai Jiao Tong University).^[^ ^23^ ^]^ The isolated cells were cultured using DMEM supplemented by 10% FBS and 40 ng mL^−1^ macrophage colony stimulating factor. After 7 days, BMDM were stimulated to M1 phenotype by lipopolysaccharide (50 ng mL^−1^), or to M2 phenotype interleukin‐4 (IL‐4, 20 ng mL^−1^), respectively.

### Cell Viability

The biocompatibility of TA/Fu‐TS was assessed using HSF and BMDM. Briefly, cells were seeded in 96‐well plates and treated with TA/Fu‐TS for 24 h. Then, viability was measured using a CCK‐8, and expressed as a percentage normalized to the value of Control.

### Cellular Uptake

HSF and BMDM were seeded in glass bottom plates, respectively. After 24 h, cells were incubated with medium containing fluorescently labeled TA/Fu‐TS for 2 and 6 h. Then, the cellular uptake was evaluated by CLSM (SP5, Leica, Germany, 358/461 nm, 466/539 nm). Furthermore, HSF and BMDM were seeded in 6‐well plates and incubated with TA/Fu‐TS for 6 h. The cellular uptake was also examined using flow cytometry, and the details were observed using TEM after standard sample preparation.

### Macrophage Phenotype‐Switching In Vitro

BMDMs were seeded on coverslips and stimulated to M1 phenotype. After treated with different formations for 24 h, BMDMs on coverslips were incubated with CD68 (The markers of macrophages) and Arj‐1 (The markers of M2 phenotype) using standard operation protocols, and then analyzed by CLSM.

### Inflammation Inhibition and Fibrosis Suppression In Vitro

Inflammation inhibition was studied by detecting pro‐inflammatory cytokines (TNF‐α, IL‐1β, and IL‐6) in supernatants of BMDM, and fibrosis suppression was assessed by measuring mRNA expression levels of fibrotic biomarkers (α‐SMA, TGF‐β1, and Col I) in HSF under mono‐ and co‐culture models.^[^ ^24^ ^]^ In mono‐model, BMDM (stimulated to M1 phenotype) and HSF were seeded in 6‐well plates and treated with different formulations for 24 h. Pro‐inflammatory cytokines in the supernatants were detected using ELISA kits (R&D Systems, USA), and the mRNA expression levels of fibrotic biomarkers were detected using qPCR with standard protocol. Furthermore, the co‐culture model of BMDM (stimulated to M1 phenotype, in the upper chamber) and 3T3 (purchased from Chinese Academy of Sciences, in the lower compartment) were carried out with in Trans‐well Boyden Chambers. After treatment with formulations for 24 h, the pro‐inflammatory cytokines in the supernatants were detected using ELISA, the fibrotic biomarkers were investigated by qPCR and western blotting.

### Rabbit HS Model Construction and HS Treatment

Adult New Zealand white rabbits (Si‐Lai‐Ke, Shanghai, approved by the Animal Experimentation Ethics Committee of School of Medicine, Shanghai Jiao Tong University, SH9H‐2022‐A612‐SB) were anesthetized by an intravenous injection of pentobarbital sodium, and constructed HS models by causing four wounds on each ear (10 mm in diameter, on the ventral side, removing perichondrium). To eliminate individual differences, HSs on each ear were divided into formulation groups (TA/Fu‐TS, TA‐TS, and Fu‐TS, respectively received a topical administration of corresponding nanogels, *n* = 6) and Control group (Carbopol gels only, *n* = 6). After wound re‐epithelialization, formulations were applied to HS every 4 days for 1 month. Formulations were left on the surface of HS tissue for 6 h at every administration. Before HS excision, the rabbits were euthanized by injecting an overdose of pentobarbital sodium.

### In Vitro/Vivo Transdermal Co‐Delivery Studies

To investigate in vitro*/*vivo transdermal co‐delivery, fluorescence‐labeled formulation nanogels were applied to human HS tissues and rabbit HS models for 1, 3, and 6 h, respectively. HS tissues were then subjected to standard methods for frozen sections (10 µm), and observed by CLSM (358/461 nm, 466/539 nm). After administration, HS tissues were cut into small pieces, dialyzed for 24 h and then detect the retention of TA and 5‐Fu using HPLC. Furthermore, HS tissues pieces (1 mm^3^) were carried out standard methods for TEM sample to observe the distribution of TA/Fu‐TS in HS tissues.

### In Vivo Scar Formation Assessment

After treatment, SEI (the dermal thickness ratio between HS tissue and normal skin) was used to evaluate the changes in HS thickness.^[^ ^25^ ^]^ The treated HS tissues were harvested, fixed with paraformaldehyde, dehydrated, embedded in paraffin, and sectioned (6 µm). Masson and Sirius red staining were used in the histopathological analysis of ECM remodeling according to the manufacturer's instructions. The ratio of Col I to Col III in Sirius red staining images was quantified using ImageJ. The ultrastructural characterization of the collagen was performed by TEM.

### In Vivo Macrophage Phenotype‐Switching, Inflammation Inhibition, and Fibrosis Suppression

The treated HS tissues were prepared for sectioning. In macrophage phenotype‐switching study, the expressions of CD68 and CD206 (biomarker for M2 phenotype) were determined using fluorescence immunostaining. The expressions of inflammatory biomarkers (IL‐1𝛽, IL‐4, IL‐6, and IL‐10) and fibrotic biomarkers (α‐SMA, TGF‐β1, Col I, and Col III) were evaluated by immunohistochemical analysis. The sections were examined and immune‐positive cells were scanned and analyzed using a scanner system (ScanScope XT, Aperio, CA) and Image J.

### In Vivo Transcriptomic Study

Transcriptomic studies using RNA sequencing were performed as previously described.^[^ ^15^, ^26^ ^]^ After treatment, HS tissues (consisting of epidermis and dermis samples, taken from above the cartilage, *n* = 3) were harvested as RNA‐Seq samples, and total RNA was extracted from HS tissue using TRIzol Reagent (Invitrogen). Then RNA quality was determined by 5300 Bioanalyser (Agilent) and quantified using the ND‐2000 (NanoDrop Technologies). Library preparation and sequencing are shown in S3 (Supporting Information). DEG in the two groups was identified according to the transcript‐per‐million reads method. Gene ontology (GO) functional enrichment pathway analysis was performed using Goatools (a Python library for gene ontology analysis).

## Conflict of Interest

The authors declare no conflict of interest.

## Supporting information

Supporting Information

## Data Availability

The data that support the findings of this study are available from the corresponding author upon reasonable request.
