# Supplementary material for: Transdermal Transfersome Nanogels Control Hypertrophic Scar Formation via Synergy of Macrophage Phenotype‐Switching and Anti‐Fibrosis Effect
Source: Adv Sci (Weinh). 2023 Dec 8;11(7):2305468. doi: 10.1002/advs.202305468 (PMC10870058; doi:10.1002/advs.202305468)
Supplement: Supplementary file 1 — Supporting Information [file ADVS-11-2305468-s001.pdf]

ADVANCED SCIENCE
Open Access

Supporting Information

for *Adv. Sci.*, DOI 10.1002/advs.202305468

Transdermal Transfersome Nanogels Control Hypertrophic Scar Formation via Synergy of Macrophage Phenotype-Switching and Anti-Fibrosis Effect

Yunsheng Chen, Kun Chen, Shan Zhong, Jiaqiang Wang, Zhixi Yu, Xiyang Sun, Yue Wang, Yan Liu* and Zheng Zhang**

Supporting Information

Transdermal Transfersome Nanogels Control Hypertrophic Scar Formation *via* Synergy of Macrophage Phenotype-switching and Anti-fibrosis Effect

Yunsheng Chen†, Kun Chen†, Shan Zhong, Jiaqiang Wang, Zhixi Yu, Xiyang Sun, Yue Wang, Yan Liu*, Zheng Zhang**

Y.S. Chen, S. Zhong, J.Q. Wang, Y. Liu,
Department of Burn, Shanghai Burn Institute, Ruijin Hospital, Shanghai Jiao Tong University School of Medicine, 197 Ruijin 2nd Road, Shanghai, 200025, China. E-mail: rjliuyan@126.com (Y. Liu)

K. Chen
Department of Burn and Plastic Surgery, Beijing Children's Hospital, Capital Medical University, National Center for Children's Health, Beijing, 100045, China
Shunyi Maternal and Children's Hospital of Beijing Children's Hospital, Beijing, 101300, China

X.Y Sun
Hongqiao International Institute of Medicine, Tongren Hospital, School of Medicine, Shanghai Jiao Tong University, 1111 XianXia Road, Shanghai, 200336, China

Y. Wang
Department of Ear Reconstruction, Plastic Surgery Hospital, Chinese Academy of Medical Sciences and Peking Union Medical College, 33 Badachu Road, Beijing, 100144, China. mail: wangyueplastic@163.com (Y. Wang)

Z.X. Yu and Z. Zhang
Department of Plastic and Reconstructive Surgery, Shanghai Ninth People's Hospital, School of Medicine, Shanghai Jiao Tong University, 639 Zhizaoju Rd, Shanghai, 200011, China. E-mail: Zhangyixin6688@163.com (Z. Zhang)

† These authors contributed equally to this work.

S1 The preparation of nanogels

The carbopol gel matrix was prepared at first as follows: (a) 1.6 g of carbopol 930 and 4 mL of propanetriol were added to 42 mL of double-distilled water; (b) the mixture was stirred for 2 h at room temperature, and then neutralized to pH 7.4 by using 2.5 mL of triethanolamine; (c) carbopol gel matrix was obtained after hydrating overnight. The nanogel formulations (TA/Fu-TS, TA-TS and Fu-TS) were prepared by solution formulations mixing with carbopol gel matrix (1:1, v/v) in a sealed container at 500 rpm.

S2 HPLC determinations

The amount of TA and 5-Fu in the receptor was determined by HPLC, respectively. The mobile phase for TA assay consisted of acetonitrile and distilled water (60:40, v/v) with monitoring at a UV absorption wavelength of 240 nm. For 5-Fu, the mobile phase was constituted of PBS (pH 3.5) and methanol (95:5, v/v) and UV absorption wavelength was 265 nm.

S3 Library preparation and Sequencing

RNA purification, reverse transcription, library construction and sequencing were performed at Shanghai Majorbio Bio-pharm Biotechnology Co., Ltd. (Shanghai, China) according to the manufacturer’s instructions (Illumina, San Diego, CA). The RNA-seq transcriptome librariy was prepared following Illumina® Stranded mRNA Prep, Ligation from Illumina (San Diego, CA) using 1μg of total RNA. Shortly, messenger RNA was isolated according to polyA selection method by oligo(dT) beads and then fragmented by fragmentation buffer firstly. Secondly double-stranded cDNA was synthesized using a SuperScript double-stranded cDNA synthesis kit (Invitrogen, CA) with random hexamer primers (Illumina). Then the synthesized cDNA was subjected to end-repair, phosphorylation and ‘A’ base addition according to Illumina’s library construction protocol. Libraries were size selected for cDNA target fragments of 300 bp on 2% Low Range Ultra Agarose followed by PCR amplified using Phusion DNA polymerase (NEB). After quantified by Qubit 4.0, paired-end RNA-seq sequencing library was sequenced with the NovaSeq 6000 sequencer (2 × 150bp read length).

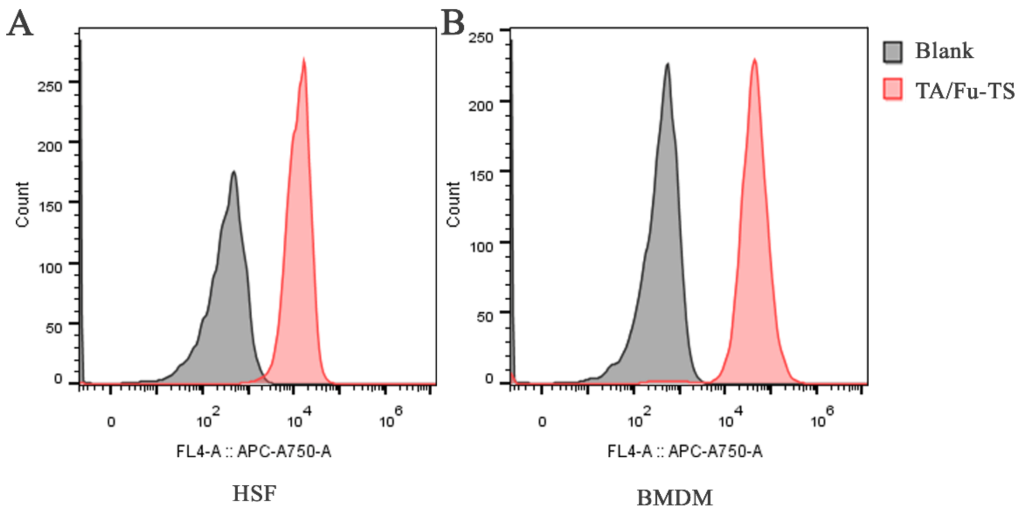

Figure S1 Flow cytometer assay of cellular uptake.

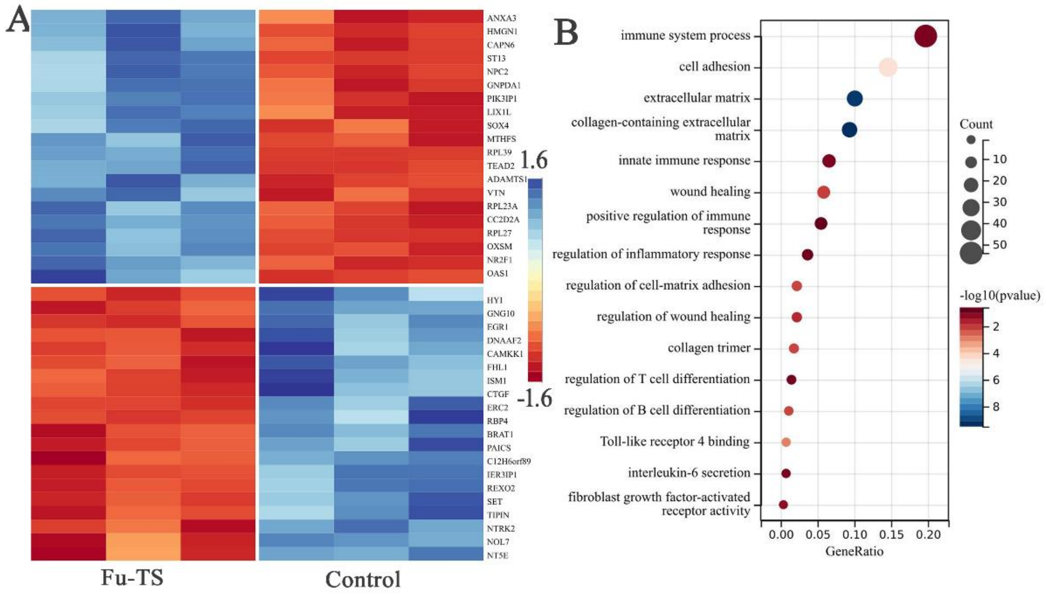

Figure S2 A: Heatmap of the top inflammation-related DEGs after being further screened (HS tissues as RNA-seq samples, n=3); **B**: GO of the DEGs showing the enriched mainly altered molecular events.
